# A qualitative burden of disease study in patients with invasive *Escherichia coli* disease aged ≥ 60 years in the United States

**DOI:** 10.1186/s12879-025-11628-5

**Published:** 2025-10-31

**Authors:** Mark A. Schmidt, Inga Gruß, James V. Davis, Judy Donald, Kelly McQuarrie, Michal Sarnecki, Joachim Doua, Luis Hernandez Pastor, Jeroen Geurtsen, Cristina Valencia, Elvira Carrió, Thomas Verstraeten, Antoine C. El Khoury

**Affiliations:** 1 Kaiser Permanente Center for Health Research, Portland, OR USA; 2 Johnson & Johnson Research & Development, Horsham, PA USA; 3 Johnson & Johnson Vaccines, Bern, Switzerland; 4 Infectious Diseases & Vaccines, Johnson & Johnson Research & Development, Beerse, Belgium; 5 Bacterial Vaccines Discovery & Early Development, Johnson & Johnson Vaccines & Prevention B.V., Leiden, Netherlands; 6 P95 Pharmacovigilance and Epidemiology Services, Leuven, Belgium; 7 Johnson & Johnson Global Services, Raritan, NJ USA

**Keywords:** *Escherichia coli*, Patient-reported outcomes, Quality of life, Bacteremia, Invasive *E. coli* disease (IED)

## Abstract

**Background:**

Invasive *Escherichia coli* disease (IED) comprises a diverse range of severe infections caused by *E. coli*, mainly affecting neonates and older adults, and is associated with severe and sometimes fatal outcomes. This study aimed to evaluate signs and symptoms associated with IED, and to assess the impact of IED on health-related quality of life (HRQoL) among older patients. Furthermore, we examined how generic patient-reported outcomes questionnaires capture experiences of patients with IED.

**Methods:**

A cross-sectional qualitative study consisting of semi-structured interviews was conducted. Participants aged ≥ 60 years who met the IED clinical case definition were enrolled within 2 weeks after the disease episode and asked to describe IED signs and symptoms and HRQoL impact through open-ended questions. The interview transcripts were analyzed using NVivo 12 and a conceptual model was built. The concepts for impacts identified by participants with IED were then compared with the concepts routinely assessed by two generic HRQoL instruments: the 36-Item Short Form Survey (SF-36) and EuroQol 5-Dimension (EQ-5D).

**Results:**

Of 80 patients screened, 43 met inclusion criteria and 16 consented to participate in the study. The interviews showed that IED affected daily routines and activities (*n* = 13), ability to move and sleep (both *n* = 11), and caused exhaustion (*n* = 10) as well as constant pain (*n* = 9). Most HRQoL impacts identified were also measured in SF-36 and EQ-5D, but concepts specific for patients with IED, not measured in SF-36 and EQ-5D, were also recognized.

**Conclusions:**

IED was shown to cause substantial physical, social, and psychological impacts in patients. This burden was especially apparent in review of patient self-reports for concepts that are part of generic HRQoL instruments (SF-36, EQ-5D), and patients also frequently reported neurological/mental impacts. Further, this study provides valuable qualitative data that may be useful for future research in developing a disease-specific instrument for the assessment of HRQoL in patients with IED.

**Supplementary Information:**

The online version contains supplementary material available at 10.1186/s12879-025-11628-5.

## Background

*Escherichia coli* are gram-negative coliform bacteria that commonly colonize the gastrointestinal tract of humans, normally without adverse consequences [1]. However, some *E. coli* strains are pathogenic and can cause enteric/diarrheagenic or extraintestinal infections, including urinary tract infections (UTIs), bacteremia, and sepsis. *E. coli* strains causing infections outside of the gastrointestinal tract are referred to as extraintestinal pathogenic *E. coli* (ExPEC) [2–4]. Moreover, gram-negative bacteria, including *E. coli*, are highly adaptive pathogens that can develop resistance to antimicrobials through several mechanisms, complicating treatment and leading to more serious disease [5]. ExPEC represents the most common gram-negative pathogen globally [6, 7] and is responsible for the development of invasive *E. coli* disease (IED), which manifests as an acute illness consistent with a systemic bacterial infection that is microbiologically confirmed by the isolation and identification of *E. coli* from a normally sterile body site, including blood, or from urine in patients with urosepsis and no other identifiable source of infection [4]. Disease severity can be stratified by the presence of bacteremia, with bloodstream infection indicating a more severe form compared to non-bacteremic cases. As society ages, IED has been a rising cause of bacteremia, sepsis, septic shock, and death [8–10]. Among adults aged > 60 years [7, 11], the incidence of *E. coli* bacteremia [12] is high and increases from 110 per 100,000 person-years among those aged 60–69 years to 319 per 100,000 person-years among those aged ≥ 80 years [13–15]. Moreover, exacerbated by the continued emergence of drug- and multidrug-resistant strains [7, 16], IED is associated with considerable hospital costs and medical resource use [17], altogether asserting the need for preventive interventions [18–20].

Although several studies report IED incidence rates and associated morbidity and mortality, the impact of IED on health-related quality of life (HRQoL) has not been evaluated. From evidence observed in patients with septic shock, severe sepsis, and bacteremia, clinical outcomes have been shown to substantially impact HRQoL, especially among females, older adults, and those with underlying medical conditions [21–23]. Although HRQoL has been reported to improve after hospital discharge, the disease sequelae are long lasting, as physical component scores, measuring the physical health status, in former sepsis patients remain lower (i.e., worse) than pre-admission levels [24] and the age-matched general population [24, 25]. Patient-reported outcome (PRO) instruments such as the 36-Item Short Form Survey (SF-36) and EuroQol 5-Dimension (EQ-5D) questionnaires have been used to assess HRQoL of patients with bacteremia and sepsis [26]. However, these instruments are generic, and their validity for measuring impact on HRQoL in patients with IED has not been established.

Here, we aimed to explore IED signs and symptoms and the impact on HRQoL in older adult patients with community-acquired IED in the United States. By comparing participants’ responses to interview prompts based on established concepts included in the SF-36 and EQ-5D HRQoL instruments (e.g., physical, psychological, social), we sought to assess the overlap with participant responses and potentially identify alternative concepts important to consider.

## Methods

### Study design, participants, and setting

We conducted a cross-sectional qualitative study consisting of semi-structured telephone interviews. The study population comprised individuals with IED, according to a pre-specified clinical case definition, as abstracted from the electronic health record (EHR) system of Kaiser Permanente Northwest (KPNW), a large, integrated healthcare delivery system serving > 630,000 individuals in Northwest Oregon and Southwest Washington in the United States. The study was approved by the Kaiser Permanente Center for Health Research institutional review board, with explicit consent to access EHR data for this study not being required.

Between July and September 2020, an automated approach was used to identify KPNW members aged ≥ 60 years with potential IED, based on electronic medical chart abstraction. In the absence of a specific ICD-10 code for invasive IED, research staff conducted a manual review of electronic health records to identify cases meeting our predefined clinical case definition [27]. Identified cases were subsequently stratified by the presence or absence of a positive blood culture. Bacteremic IED cases were defined as those in which *E. coli* infection was microbiologically confirmed from the blood and the participant experienced the presence of any of the following symptoms: temperature < 36 °C or > 38 °C, tachycardia (heart rate > 90 beats per min), tachypnea (respiratory rate > 20 breaths per min), or white blood cell (WBC) count < 4 or > 12 × 10^9^/L or 10% immature (band) forms. Non-bacteremic IED cases were defined as those presenting *E. coli* in the urine (≥ 10^5^ colony-forming units per mL) with no other identifiable site of infection and with either an acute change in total Sequential Organ Failure Assessment (SOFA) score of ≥ 2 points above baseline (i.e., the SOFA score on the first day of hospitalization) or with UTI signs and symptoms and meeting systemic inflammatory response syndrome criteria [28]. Individuals with positive cultures from other sterile sites (alone or in combination with positive blood cultures) or blood cultures with mixed pathogens (with the presence of *E. coli*) were excluded from the study. Individuals treated for community-acquired IED in the outpatient setting and inpatient setting were eligible.

Eligible participants were contacted within 2 weeks after the end of their IED episode, defined as 7 days after the date of the last outpatient encounter for that episode of illness or, for hospitalized patients with IED, as the date of discharge from the hospital.

### Data collection

After prior oral informed consent, the interviews took place by telephone by a researcher not involved in patient care. Only eligible participants were allowed to provide responses; caregivers could not assist the participants or provide responses on behalf of the participants. Consent was reconfirmed before initiation of the interview. The interview guide consisted of probing questions in two parts and is described in full in Additional file 1. The first part collected information on the description of signs and symptoms experienced during the IED episode at three stages: before hospitalization (–45 to − 6 days), immediately before and during hospitalization (–5 days to discharge), and after discharge (from discharge to time of interview [maximum 14 days after discharge]). The second part collected information on the impact of IED on HRQoL. Participants were asked to provide comments using their own language, and prompt questions were used to clarify answers when necessary. The prompts were based on concepts from the generic HRQoL instruments (SF-36 and EQ-5D). All interviews were audio recorded.

Data from medical records were collected to characterize study participants and determine case definitions. UTI cases were defined as those with an ICD code indicative of a coinciding UTI within 10 days from the IED diagnosis date. The source of the IED was determined through review of clinical notes or laboratory-confirmed documentation of infection, and was defined as the occurrence of UTI, pneumonia, cholecystitis, gastroenteritis, meningitis, abscess, surgical site infection, or pleuritis within the 10-day period preceding the IED index date.

### Data analysis

All participant interview transcripts were de-identified for analysis and reporting using a unique study identification number generated at the time of study entry. Transcripts were coded and analyzed with NVivo 12 [29]. A template analysis [30] was conducted to identify disease symptoms, HRQoL concerns, and feedback from the participants and to develop a conceptual framework. The coding template contained the following codes: HRQoL concerns; disease symptoms with the codes before, during, and after hospitalization; and feedback (only applied to the last eight transcripts to capture feedback about the conceptual model). Two researchers applied the coding template to all transcripts. A theoretical model grounded in the data was created to summarize findings.

### PRO measurement instrument comparison

Impacts reported in the interviews were compared with concepts assessed in the generic HRQoL instruments (SF-36 v1 and EQ-5D). The SF-36 includes eight concepts that measure physical functioning, role limitations due to physical health problems, bodily pain, general health, vitality, social functioning, role limitations due to emotional problems, and mental health. The EQ-5D questionnaire includes five concepts: mobility, self-care, usual activities, pain/discomfort, and anxiety/depression. Each concept has three response levels of severity: no problems, some problems, and extreme problems [31].

### Data management

Participation in the study was confidential and all data were de-identified and handled and stored following state and federal data protection laws.

## Results

### Enrollment

**Fig. 1:**
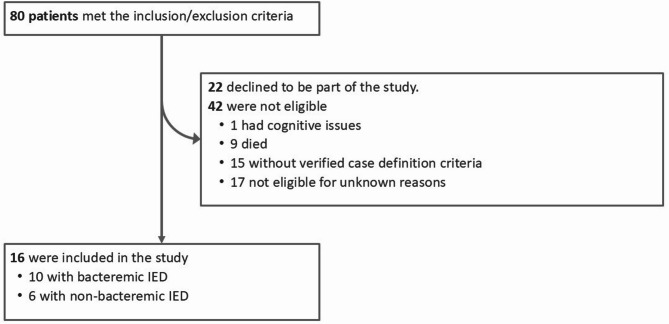
Flow diagram of participants’ enrollment Seventeen participants were excluded as unknown because they did not meet the inclusion criteria anymore or had a diagnosis of dementia

Between July and November 2020, 80 patients were screened, of whom 43 met the specified inclusion criteria and were invited to participate in the study (Fig. 1). Sixteen patients consented to participate and were enrolled; ten had bacteremic IED and six had non-bacteremic IED. The 16 study participants had a similar age range and sex distribution as the 27 patients who refused to participate (data not shown).

### Clinical and demographic characteristics

Fourteen of the 16 study participants were hospitalized. Review of medical records found that UTI was the most frequent source of infection (14 of 16), followed by gastroenteritis, abscess, and pneumonia (one each), including one participant with both UTI and pneumonia. Sepsis was frequent among participants, affecting nine of ten participants with bacteremic IED and four of six participants with non-bacteremic IED.

Demographics and duration of hospitalization stay did not differ substantially between participants with bacteremic and non-bacteremic IED (Table 1, Additional file 2, Table S1**)**. Of the ten bacteremic participants, eight were female and two were male, with a median hospitalization duration of 4 days (range, 3–5 days) and an age range of 61–90 years. Of the six participants with non-bacteremic IED, four participants were female and two were male, with a median hospitalization duration of 5 days (range, 2–5 days) and an age range of 68–85 years. Clinical characteristics differed between participants with bacteremic and non-bacteremic IED. UTI, pyelonephritis (UTI affecting one or both kidneys), and tachypnea occurred more frequently in those with non-bacteremic IED (Table 1).

**Table 1:** Demographic and clinical characteristics of study participants with bacteremic and non-bacteremic IED

Participants	Total *N* = 16	Bacteremic *n* = 10	Non-bacteremic *n* = 6
Age			
Mean (range)	75.25 (61–90)	75.2 (61–90)	75.33 (68–85)
Sex,* n* (%)			
Female	12 (75)	8 (80)	4 (67)
Male	4 (25)	2 (20)	2 (33)
Met case definition criteria			
UTI diagnosis^1^, *n* (%)	11 (69)	5 (50)	6 (100)
Urethritis, *n* (%)	10 (63)	7 (70)	3 (50)
Pyelonephritis, *n* (%)	3 (19)	0	3 (50)
Temperature < 36 °C or > 38 °C, mean (range), *n* (%)	39.2 (38.2–41.7), 9 (56)	39 (38.2–39.4), 5 (50)	39.5 (38.6–41.7), 4 (67)
Tachycardia (HR) > 90 bpm, mean (range), *n* (%)	115.17 (104–141), 6 (38)	113.33 (112–115), 3 (30)	117 (104–141), 3 (50)
Tachypnea (RR > 20 breaths per min), mean (range), *n* (%)	29 (22–41), 3 (19)	0	29 (22–41), 3 (50)
White blood cell count < 4 or > 12 × 10^9^/L, mean (range), *n* (%)	18.29 (12.19–28.5), 13 (81)	18.53 (12.19–28.5), 9 (90)	17.75 (13.97–20.37), 4 (67)
10% immature (band) forms, mean (range), *n* (%)	21.8 (21.8–21.8), 1 (6)	21.8 (–), 1 (10)	0 (–), 0
Sepsis, *n* (%)	13 (81)	9 (90)	4 (67)
Source of IED^2^, *n *(%)			
UTI	14 (88)	8 (80)	6 (100)
Gastroenteritis	1 (6)	1 (10)	0
Abscess	1 (6)	1 (10)	0
Pneumonia	1 (6)	0	1 (17)
Hospitalization			
Hospital days,^3^ mean (range), *n* (%)	3.93 (2–5), 14 (88)	3.78 (3–5), 9 (90)	4.2 (2–5), 5 (83)
Outpatients			
Emergency department visit, *n* (%)	2 (13)	1 (10)	1 (17)

*bpm* beats per min, *HR* Heart rate, *IED* Invasive *Escherichia coli* disease, *RR* Respiratory rate, *UTI* Urinary tract infection

^1^UTI cases were defined as those with an ICD code indicative of a coinciding UTI within 10 days from the IED diagnosis date

^2^The source of the IED was determined through review of clinical notes or laboratory-confirmed documentation of infection, and was defined as the occurrence of UTI, pneumonia, cholecystitis, gastroenteritis, meningitis, abscess, surgical site infection, or pleuritis within the 10-day period preceding the IED index date

^3^Hospitalizations were shorter than usually due to COVID-19 and the efforts around limiting the number of hospitalization days

### Perspectives of participants with IED

#### Descriptions of signs and symptoms

Symptoms preceding the IED diagnosis (–45 to − 5 days) were reported by six participants (two bacteremic participants and four non-bacteremic participants). Among these symptoms, burning sensation when urinating and pain or pressure in the pelvic area or lower abdomen were more frequently described by participants with bacteremic IED; pain or pressure in the pelvic area or lower abdomen, shortness of breath, back pain, and swelling in the left arm were more frequently described by participants with non-bacteremic IED (Table 2, Additional file 2, Table S2). The remaining ten participants did not report symptoms prior to IED diagnosis.

**Table 2:** Signs and symptoms reported by study participants with bacteremic and non-bacteremic IED

Participants with IED signs and symptoms, *n*	Total	Bacteremic	Non-bacteremic
	*N* = 16	*n* = 10	*n* = 6
Symptoms preceding IED diagnosis (–45 to − 5 days)	6	2	4
Pain or pressure in pelvic area or lower abdomen	3	1	2
Shortness of breath	2	0	2
Back pain	1	0	1
Swelling in left arm	1	0	1
Burning sensation when urinating	2	2	0
Symptoms immediately before and during hospitalization (–5 days to discharge)	16	10	6
Intense chills/shaking	15	10	5
Nausea/dry heaving	13	9	4
Fever/elevated temperature	12	6	6
Loss of appetite	9	6	3
Weakness/fatigue	13	9	4
Flank/stomach pain	8	5	3
Diarrhea	8	6	2
Shortness of breath/low oxygen levels	8	4	4
Tingling/burning during urination	4	3	1
Back pain	2	0	2
Low blood pressure symptoms	2	2	0
Headache	3	3	0
Lightheadedness	3	2	1
Inability to urinate/empty bladder	6	3	3
Constipation	3	1	2
Foul-smelling urine	1	0	1
Feeling of imbalance	2	1	1
Blood in urine	4	1	3
Frequent urination	2	0	2
Ongoing symptoms after hospital discharge (discharge to interview [maximum 14 days])	11	8	3
Weakness/fatigue	9	8	1
Diarrhea	3	3	0
Loss of appetite	5	4	1
Tingling/burning during urination	2	1	1
Stomach pain	1	1	0
Constipation	1	0	1
Signs and symptoms – representative quotes			
Pain and dry heaving			
*“That would be the flank pain and dry heaving. Those were the only two things that were going on.”* – Participant #11 (UTI, sepsis [bacteremic IED])			
*“I woke up in the early AM*,* like 4:00 AM with pretty significant pain under my left rib cage. And then I started dry heaving for hours.” –* Participant #1 (UTI, sepsis [bacteremic IED])			
Nausea and dry heaving			
*“First I couldn’t eat breakfast or dinner. And then I was throwing up by the evening.”* – Participant #13 (UTI, sepsis [bacteremic IED])			
*“He asked me some questions I said I can’t answer you unless we stop this nausea and vomiting*,* I said I can’t talk to you.” ****–*** Participant #6 (UTI, sepsis [bacteremic IED])			
Diarrhea			
*“The diarrhea*,* you didn’t know. It actually filled your pants before you even knew it. You could be asleep in bed and you get up and the diaper is full.” ****–*** Participant #12 (UTI, sepsis [bacteremic IED])			
“I had had inflammatory bowel syndrome symptoms of abdominal pain which didn’t go away. Everything I did, didn’t go away, didn’t go away.” – Participant #10 (UTI [non-bacteremic IED])			
Intense chills/shaking and weakness/fatigue			
*“I would say the uncontrollable shakes in my entire body. I would say the weakness. (long pause) I don’t know if I would say sweating or sweating and chills. Maybe I would say that also.”* – Participant #14 (Abscess [bacteremic IED])			
*“I spent the entire day in the ER with them giving me IVs and what have you. I landed there because I was having chills and fever.” –* Participant #1 (UTI, sepsis [bacteremic IED])			
“*I was cold all the time but it wasn’t chills. I just was cold and had a hard time getting warm. They would bring me blankets but it was hard to get warm.*” – Participant #16 (UTI, sepsis [non-bacteremic IED])			
*“Until I was laying on the couch Sunday night and all of a sudden*,* I got the chills and the shakes. I guess my blood pressure and sats dropped*,* I went to the hospital so real sudden onset.”* – Participant #15 (UTI, pneumonia, sepsis [non-bacteremic IED])			

Quotes are shown in italics; cause of IED, presence of sepsis and bacteremic status are indicated in brackets

*ER* Emergency room, *IED* Invasive *Escherichia coli* disease, *IV* Intravenous (therapy), *UTI* Urinary tract infection

All study participants reported symptoms immediately before and during hospitalization. The most frequently reported symptoms were intense chills/shaking, nausea/dry heaving, fever (more frequently in non-bacteremic than in bacteremic patients), and weakness/fatigue. Additionally, participants with non-bacteremic IED reported shortness of breath. Overall, most participants experienced symptoms related to the urinary system (tingling/burning during urination, inability to urinate/empty bladder, foul-smelling urine, blood in urine, and frequent urination).

Health complaints after hospital discharge were more often reported by participants with bacteremic IED (17 complaints in ten patients) than by participants with non-bacteremic IED (four complaints in six patients). Weakness/fatigue, loss of appetite, and diarrhea were complaints described by participants with bacteremic IED, whereas weakness/fatigue, loss of appetite, tingling/burning during urination, and constipation were complaints described by participants with non-bacteremic IED. Representative quotes reflecting participants’ experiences regarding IED signs and symptoms are reported in Table 2.

#### IED impact on HRQoL

Psychological impacts were reported frequently. Participants mostly experienced fear and confusion, anxiety, depression, and disorientation, while some participants felt frustration (Table 3, Additional file 2, Table S3). These impacts were more often described by participants with bacteremic IED than by participants with non-bacteremic IED.

**Table 3:** IED psychological, physical, and social impacts reported by study participants with bacteremic and non-bacteremic IED

Frequency of reported impacts, *n*	Total *N* = 16	Bacteremic *n* = 10	Non-bacteremic *n* = 6
Psychological impacts	10	8	2
Confusion	5	5	0
Anxiety	3	2	1
Depression	3	2	1
Frustration	2	2	0
Disorientation	3	3	0
Fear	7	6	1
Physical impacts	16	10	6
Ability to engage in mobility	11	8	3
Impact on daily routines and activities	13	9	4
Not being able to sleep	11	6	5
Inability to communicate	0	0	0
Inability to feel balanced/imbalance	4	2	2
Learning to live with constant pain	9	5	4
Increased exhaustion	10	5	5
Not being able to concentrate	2	1	1
Social impacts	14	8	6
Engage in fewer activities	14	8	6
Temporarily rely on the support of caregiver/family member/friend	3	2	1
**Impacts – representative quotes**			
Mobility			
*“One of the things I noticed was before I went to the hospital*,* if I’d be laying in bed and try to sit up and swing my legs over the side of the bed I had a really difficult time doing that.”****–*** Participant #1 (UTI, urethritis [bacteremic IED])			
*“The ordinary getting up and down and running around seemed more difficult”* Participant #6 (UTI and urethritis [bacteremic IED])			
Pain			
*“Well*,* I haven’t been going walking just because I’ve been feeling pain. So that is probably contributing to why I’m getting tired so easily.” ****–*** Participant #1 (UTI, urethritis [bacteremic IED])			
*“Oh my gosh! I couldn’t function*,* the pain was so intense… I just wanted to be out of pain.”****–*** Participant #3 (UTI, pyelonephritis [non-bacteremic IED])			
Exhaustion			
*“I never felt that weak before or a lot of fatigue and tired*,* tired.”* – Participant #4 (Gastroenteritis [bacteremic IED])			
*“Yeah I was really tired and I just wanted to sleep. I couldn’t.”* – Participant #3 (UTI, pyelonephritis [non-bacteremic IED])			
Anxiety			
*“Of course*,* I had a lot of anxiety because I didn’t really know what was going on because of the confusion I guess.” *– Participant #6 (UTI and urethritis [bacteremic IED])			
*“A lot of anxiety*,* because I want to know what causes it.”* – Participant #2 (UTI, pyelonephritis [non-bacteremic IED])			
Daily activities			
Walking: *“Well*,* I haven’t been going walking just because I’ve been feeling pain. So that is probably contributing to why I’m getting tired so easily.”* – Participant #1 (UTI, urethritis [bacteremic IED])			
Shopping and meetings: *“I have to go to the grocery store*,* anyhow they took me*,* I had to go get my medications three times*,* every third day. So I had my children take me to all my doctor appointments.”* – Participant #5 (UTI, urethritis [bacteremic IED])			
Eating: *“But I’ve had a lot of issues with not wanting to hear food*,* see food*,* smell food*,* eat food.”* – Participant #1 (UTI, urethritis [bacteremic IED])			
Cooking: *“My kids would bring food for maybe that first week only as I was not able to do my usual cooking.”* – Participant #5 (UTI, urethritis [bacteremic IED])			
Overall			
*“So overall*,* my condition is obviously much worse than it was when I was pre-hospitalization.”* – Participant #14 (Abscess [bacteremic IED])			
*“I just can’t function like I usually do. I can’t get as much done.”* – Participant #16 (UTI, pyelonephritis [non-bacteremic IED])			
*“It happened so quickly. I went from being absolutely normal the day before to that morning being violently ill. It just was like just so fast…”* ***–*** Participant #3 (UTI, pyelonephritis [non-bacteremic IED])			

Quotes are shown in italics; cause of IED, presence of sepsis and bacteremic status are indicated in brackets

*IED* invasive *Escherichia coli* disease, *UTI* Urinary tract infection

All participants reported that IED impacted their physical functioning. The disease mostly affected their daily routines and activities, their ability to move and sleep, and caused them exhaustion and constant pain. One participant with bacteremic IED and one participant with non-bacteremic IED described difficulties with concentration.

**Fig. 2:**
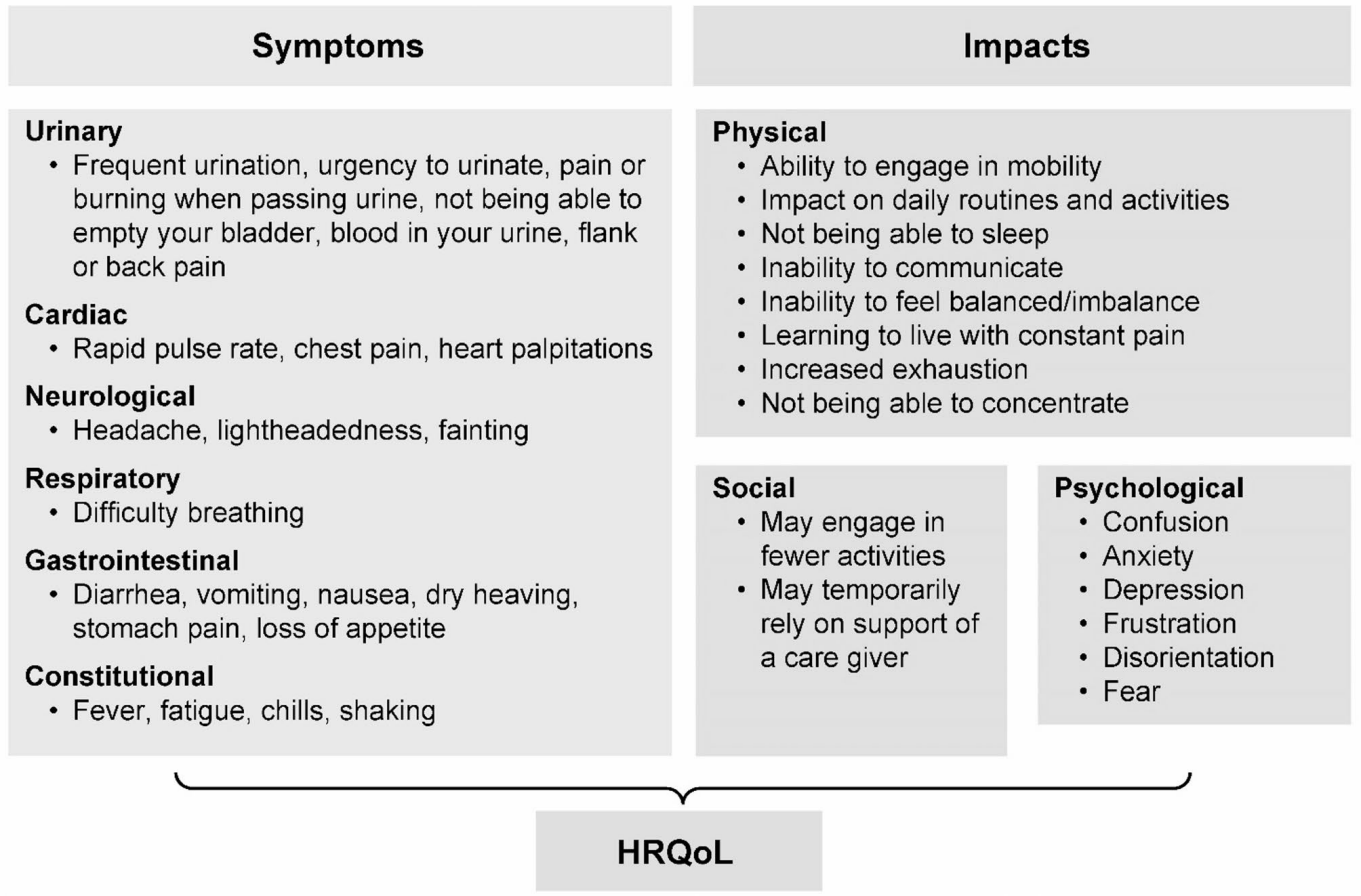
Conceptual framework of perspectives from participants with IED. *HRQoL* health-related quality of life, *IED* invasive *Escherichia coli* disease

Overall, almost all study participants reported that the disease impacted their social life by limiting their capacity to engage in social activities. Temporary support of caregivers, family members, or friends was required by some participants. Representative quotes reflecting how IED has impacted their lives were extracted from the transcripts and are reported in Table 3. The symptoms and impacts reported by the study participants were categorized, and a conceptual framework was developed to show their impact on their HRQoL (Fig. 2). As the differences between bacteremic and non-bacteremic participants seemed limited, a single conceptual framework was considered to be sufficient.

### Comparing interview outcomes with the SF-36 and EQ-5D questionnaires

The impacts reported by the study participants included in the conceptual framework were compared with those included in two generic PRO instruments, the SF-36 and EQ-5D (Table 4). For six psychological impacts included in the model, SF-36 covered four (“confusion,” “anxiety,” “depression,” and “frustration”), and the EQ-5D covered two (“anxiety” and “depression”). For the eight physical impacts in the model, both instruments included the following concepts: “mobility,” “daily routines and activities,” and “learning to live with constant pain,” whereas SF-36 additionally included the concept of “increased exhaustion” as a possible physical impact. In addition, both PRO instruments included one of the two social impacts observed: the capacity to “engage in fewer activities.” Impacts identified in this study, with no direct measure in the PRO questionnaires, were mostly mental or neurological, namely “disorientation,” “fear,” “not being able to sleep,” “inability to communicate,” “inability to feel balanced/imbalance,” and “not being able to concentrate.” Additionally, “temporarily relies on the support of caregiver/family member/friend” was also absent in both instruments.

**Table 4:** Reported Move Table 4 down to sit with the section (comparing interviews) below IED impacts that are concepts assessed in the SF-36 and EQ-5D

IED impacts learned in this study	Concepts assessed in SF-36 questionnaire		Concepts assessed in EQ-5D questionnaire
Psychological impacts			
Confusion	Yes		No
Anxiety	Yes		Yes
Depression	Yes		Yes
Frustration	Yes		No
Disorientation	No		No
Fear	No		No
Physical impacts			
Ability to engage in mobility	Yes		Yes
Impact on daily routines and activities	Yes		Yes
Not being able to sleep	No		No
Inability to communicate	No		No
Inability to feel balanced/imbalance	No		No
Learning to live with constant pain	Yes		Yes
Increased exhaustion	Yes		No
Not being able to concentrate	No		No
Social impacts			
Engage in fewer activities	Yes		Yes
Temporarily rely on the support of caregiver/family member/friend	No		No

*EQ-5D* EuroQol 5-Dimension, *IED* Invasive *Escherichia coli* disease, *SF-36* 36-Item Short Form Survey

## Discussion

Patients’ perspectives are essential for a full and complete understanding of the signs and symptoms of disease, and associated impacts on HRQoL. Through concept elicitation interviews with older adult patients, this study assessed for the first time the perspective of participants with recent IED on the signs and symptoms of the disease and how this impacts perceived HRQoL. Based on this, a conceptual framework for how patients experienced IED was developed and subsequently compared with the concepts included in two generic PRO instruments.

IED frequently led to signs and symptoms immediately before and during hospitalization (for those patients who were hospitalized). At that stage, the most frequently experienced symptoms were constitutional (intense chills/shaking, weakness/fatigue), gastrointestinal (nausea/dry heaving), and/or related to the genitourinary tract. Symptoms prior to the IED diagnosis were not frequent, potentially reflecting a fast onset and worsening of disease. In those who experienced early symptoms, pain or pressure in the pelvic area or lower abdomen was most often described. After hospital discharge, most patients with IED experienced ongoing weakness and fatigue, and some lost their appetite or suffered diarrhea. Overall, the health complaints described align with the previous reports of IED being a severe disease, with the urinary tract as an important source of infection [11, 28].

The symptoms of IED had a physical impact on patients’ lives, frequently coinciding with social and psychological impacts. In general, mobility issues impeded daily routines and activities, and sleep difficulties, pain, and increased exhaustion were the physical impacts most often experienced. These findings are consistent with results from studies conducted among patients with sepsis, where the physical component was found to be most affected by the disease [21–23]. The disease affected the social life of most patients with IED (i.e., all participants with non-bacteremic IED and most of the participants with bacteremic IED) by reducing their capacity to engage in activities. Psychological impacts due to IED were also described by most patients with IED.

The IED impacts identified were broadly assessed by interview prompts based on concepts evaluated in two generic PRO instruments, the SF-36 and EQ-5D questionnaires, indicating that the concepts included in these instruments may be useful for assessing HRQoL of patients with IED. Previously, those questionnaires have been used directly in studies of sepsis and septic shock, demonstrating their ability to evaluate the impact of disease [21, 23, 32–34]. However, some impacts of IED observed in our study, such as “fear,” “disorientation,” and “not being able to concentrate” were not included in the concepts assessed by the generic PRO questionnaires, suggesting that using additional PRO questionnaires specialized in specific neurological/mental outcomes, such as the Neuro-QoL questionnaire [35] and AQoL 8D [36] for evaluating cognitive capacities and psychosocial aspects of the quality of life, may be considered for further evaluations of the impact of IED on patients’ HRQoL. Also, the recently developed EQ Health and Wellbeing tool, designed to complement EQ-5D, might be useful to capture the impact of IED on patients’ wellbeing [37].

This study has several limitations. The study population was small; 16 participants were not representative of the entire IED patient population. Additionally, the interviews were conducted in 2020. Furthermore, patients’ perceptions from KPNW members in the United States might not be generalizable to other populations and countries. Larger studies must be done in several geographic regions (likely involving different health systems) to confirm our findings. After review of medical records, UTI was the most frequent source of infection, which may limit generalizability of these results to other sources of infection. Participants were mostly female, which might have biased some of the reported outcomes, as women and men may perceive and describe IED symptoms and related impacts differently, although no substantial differences were observed between sexes in this study. The SOFA baseline was assessed on day 1 of hospitalization. This may have introduced selection bias, as a further increase in SOFA score, and therefore worsening of the disease, is required. It is also uncertain how reliable the SOFA score is at day 1, considering that not all parameters to determine the SOFA score may be available shortly after hospitalization. Only patients with IED who were alive and physically and mentally able to complete the structured interviews without caregiver assistance were included in this study. This may have led to an underestimation of the total burden for IED and/or missing some key concepts related to the sickest patients, some of whom may have eventually died from their severe illness, and who therefore would not have been included in this assessment. While we appreciate this limitation, we preferred to rely on first-hand information, as information relayed by caregivers may not adequately reflect the patient’s perceived burden. Finally, the impact of the COVID-19 pandemic on the healthcare system might have affected the length of the hospitalizations and may have potentially influenced the assessment of the psychological and social impacts of IED.

## Conclusion

IED represents an important disease burden with substantial impact on HRQoL. The participants in this study described diverse signs and symptoms of IED throughout the disease course, especially shortly before and during hospitalization. All patients with IED experienced a physical impact due to IED, frequently accompanied by social and psychological consequences. This study supports the use of concepts from the SF-36 and EQ-5D PRO instruments to assess HRQoL of IED, with additional neurological/mental and social concepts needing to be considered. Future research should therefore focus on exploring the possibility of developing a PRO instrument specific for IED, with the aim to capture and describe the full HRQoL disease burden.

## Supplementary Information

Supplementary Material 1

Supplementary Material 2

## Data Availability

The data sharing policy of Johnson & Johnson is available at (https://www.jnj.com/policies-reports/our-position-on-clinical-trial-data-transparency). As noted on this site, requests for access to the study data can be submitted through Yale Open Data Access (YODA) Project site at (https://yoda.yale.edu).

## Abbreviations

EQ-5D: EuroQol 5-Dimension
EHR: Electronic health record
ExPEC: Extraintestinal pathogenic *Escherichia coli*
HRQoL: Health-related quality of life
IED: Invasive *Escherichia coli* disease
KPNW: Kaiser Permanente Northwest
PRO: Patient-reported outcome
SF-36: 36-Item Short Form Survey
SOFA: Sequential Organ Failure Assessment
UTI: Urinary tract infection
