# Supplementary material for: A qualitative burden of disease study in patients with invasive Escherichia coli disease aged ≥ 60 years in the United States
Source: BMC Infect Dis. 2025 Oct 31;25:1466. doi: 10.1186/s12879-025-11628-5 (PMC12577188; doi:10.1186/s12879-025-11628-5)
Supplement: Supplementary file 1 — Supplementary Material 1 [file 12879_2025_11628_MOESM1_ESM.docx]

## **Additional material 1**

**Interview guide**

Hello, my name is __________ and I am from the Kaiser Permanente Northwest Center for Health Research. Thank you for taking the time today to help us with the [study name]. Is this still a good time to talk? [If not, re-schedule]

As you might recall from the invitation letter, the Center for Health Research is working with [name client? Janssen] to learn more about symptoms patients experience when they have been diagnosed with invasive *e coli* disease. You were invited to be interviewed because you recently had a hospital stay related to this diagnosis. We would like to learn more about the symptoms you experienced and how you felt. There are no right or wrong answers to any questions. As I said, the purpose is to learn how you experienced the disease. We expect the interview to take 30–45 minutes.

I will be recording the interview to make sure I don't miss anything. Everything you tell me today will be kept confidential. In future publications we may use any quotes that explain a point particularly well, but nothing we use will identify you personally in any way. You do not have to answer any questions you don't want to, and you can stop at any time.

Do you have any questions before we get started? [answer questions]

I will start the recording and interview now.

You were recently discharged from the hospital. How are you feeling?

Can you tell me about the symptoms and experiences you had that led to your hospitalization? What were you experiencing that caused admission to the hospital? (interviewer: After the person describes the symptoms, use the following probes)

Prompts:

Did you experience

- Temperature less than 36 degrees C or temperature greater than 38 degrees C
- Chills or rigors
- Nausea
- Vomiting
- Shortness of breath
- Lightheadedness
- Rapid heart rate
- Heart palpitations (a racing, uncomfortable or irregular heartbeat or a sensation of "flopping" in the chest)
- Chest pain
- Fainting
- Frequent urination or having had to toilet very often
- Urgency to urinate
- Pain or burning when passing urine
- Not being able to empty your bladder completely
- Pain or uncomfortable pressure in the lower abdomen or pelvic area
- Low back pain caused by your urinary tract infection
- Blood in your urine
- Feeling confused
- Difficulty concentrating
- Tired or feeling fatigued

Let me summarize, you describe that you experienced the following symptoms: […]. Did I miss any?

How did these symptoms impact your life? Can you describe how they impacted your quality of life?

Prompts: feelings they may have experienced such as confusion, anxiety, depression, exhaustion, fear; impact on their ability to engage in mobility, daily routines and activities, sleep, ability to communicate

How long did you stay in the hospital?

Did you experience any new/different symptoms after your admission? Could you describe how your symptoms changed over time during your hospital stay?

Prompts: Did the symptoms get worse at first while you were in the hospital? Did you experience the same symptoms for most of the time? Were these symptoms more intense or ease up over the course of your hospital stay? Did some symptoms disappear and were replaced with other symptoms?

Have you experienced these symptoms in the past? How did your recent experience compare to past events?

Looking back at your recent illness experience, what were the three most significant symptoms you experienced? How and why were these the most impactful?

How are you feeling now? Do you continue to experience any of these symptoms? How do these symptoms currently affect your quality of life?

Any final thoughts you want to share about your recent illness experience?

We are helping a pharmaceutical company to prepare for a vaccine trial to prevent IED. We want to provide participants in this study with instructions describing symptoms that should lead them to contact the clinic and seek medical care. We would like your opinion about these instructions.

Please read out loud and I will ask you for your thoughts and opinions about the instructions.

1. What did the instructions mean to you, were they clear, do you have suggestions that would make them better?
2. What did <symptom 1> mean to you? is there a better way to describe this symptom?

<interviewer: go through each symptom with these same questions>

1. Are there symptoms that you experienced that are missing from this set of instructions?
2. Any additional thoughts or suggestions about these instructions?
3. Considering how you felt before you were hospitalized what else do you think people would want to know?
