# Supplementary material for: A qualitative burden of disease study in patients with invasive Escherichia coli disease aged ≥ 60 years in the United States
Source: BMC Infect Dis. 2025 Oct 31;25:1466. doi: 10.1186/s12879-025-11628-5 (PMC12577188; doi:10.1186/s12879-025-11628-5)
Supplement: Supplementary file 2 — Supplementary Material 2 [file 12879_2025_11628_MOESM2_ESM.docx]

## Additional material 2

#### Table S1 Demographic and clinical characteristics per study participant

| **Case definition** | **Bacteremic** | | | | | | | | | | **Non-bacteremic** | | | | | |
| --- | --- | --- | --- | --- | --- | --- | --- | --- | --- | --- | --- | --- | --- | --- | --- | --- |
| **Participant number** | 1 | 4 | 5 | 6 | 7 | 8 | 11 | 12 | 13 | 14 | 2 | 3 | 9 | 10 | 15 | 16 |
| **Age*/*sex** | | | | | | | | | | | | | | | | |
| Age | 70 | 79 | 90 | 78 | 61 | 73 | 70 | 64 | 78 | 89 | 73 | 69 | 68 | 81 | 85 | 76 |
| Sex | F | F | F | F | M | F | F | F | F | M | F | F | M | M | F | F |
| **Met case definition criteria** | | | | | | | | | | | | | | | | |
| UTI diagnosis | Yes | No | Yes | No | Yes | No | Yes | Yes | No | No | Yes | Yes | Yes | Yes | Yes | Yes |
| Urethritis | Yes | No | Yes | Yes | Yes | Yes | Yes | Yes | No | No | No | No | Yes | Yes | Yes | No |
| Pyelonephritis | No | No | No | No | No | No | No | No | No | No | Yes | Yes | No | No | No | Yes |
| Temperature < 36°C or > 38°C | wnr | wnr | wnr | 38.2 | wnr | wnr | 39.4 | 39.4 | 39.3 | 38.7 | wnr | 39 | 38.6 | wnr | 41.7 | 38.7 |
| Tachycardia (HR > 90 bpm) | wnr | wnr | 112 | wnr | wnr | wnr | wnr | 115 | 113 | wnr | 106 | wnr | 104 | wnr | wnr | 141 |
| Tachypnea (RR > 20 breaths/min) | wnr | wnr | wnr | wnr | wnr | wnr | wnr | wnr | wnr | wnr | wnr | wnr | 41 | 24 | wnr | 22 |
| White blood cell count < 4 or  > 12 × 10^9^/L | 19.38 | 28.5 | wnr | 12.19 | 20.27 | 14.35 | 18.52 | 16.33 | 19.37 | 17.87 | 17.96 | 18.7 | 20.37 | 13.97 | wnr | wnr |
| 10% immature (band) forms |  |  |  |  | 21.8 |  |  |  |  |  |  |  |  |  |  |  |
| Sepsis | Yes | Yes | Yes | Yes | Yes | Yes | Yes | Yes | Yes | No | Yes | No | Yes | No | Yes | Yes |
| **Cause of IED** | | | | | | | | | | | | | | | | |
| UTI | Yes | No | Yes | Yes | Yes | Yes | Yes | Yes | Yes | No | Yes | Yes | Yes | Yes | Yes | Yes |
| Gastroenteritis | No | Yes | No | No | No | No | No | No | No | No | No | No | No | No | No | No |
| Abscess | No | No | No | No | No | No | No | No | No | Yes | No | No | No | No | No | No |
| Pneumonia | No | No | No | No | No | No | No | No | No | No | No | No | No | No | Yes | No |
| **Hospitalization** | | | | | | | | | | | | | | | | |
| Hospital days^a^ | 3 | 4 | 3 | 4 | 3 | 4 | 4 |  | 5 | 4 | 5 | 2 | 4 | 5 | 5 |  |
| Emergency department visit | No | No | No | No | No | No | No | Yes | No | No | No | No | No | No | No | Yes |

^a^Hospitalizations were shorter due to COVID-19 and the efforts around limiting the number of hospitalization days

bpm, beats per min; F, female; HR, heart rate; IED, invasive *Escherichia coli* disease; M, male; RR, respiratory rate; UTI, urinary tract infection; wnr, within normal range

#### Table S2 IED signs and symptoms reported per study participant

| **IED case definition** | **Bacteremic** | | | | | | | | | | **Non-bacteremic** | | | | | |
| --- | --- | --- | --- | --- | --- | --- | --- | --- | --- | --- | --- | --- | --- | --- | --- | --- |
| **Participant number** | 1 | 4 | 5 | 6 | 7 | 8 | 11 | 12 | 13 | 14 | 2 | 3 | 9 | 10 | 15 | 16 |
| **Symptoms *preceding IED diagnosis* (–45 to –5 days)** | | | | | | | | | | | | | | | | |
| Pain or pressure in pelvic area or lower abdomen | X |  |  |  |  |  |  |  |  |  | X | X |  |  |  |  |
| Shortness of breath |  |  |  |  |  |  |  |  |  |  |  | X |  |  | X |  |
| Back pain |  |  |  |  |  |  |  |  |  |  |  | X |  |  |  |  |
| Swelling in left arm |  |  |  |  |  |  |  |  |  |  |  |  | X |  |  |  |
| Burning sensation when urinating | X |  |  |  | X |  |  |  |  |  |  |  |  |  |  |  |
| **Symptoms *immediately before* and *during hospitalization* (–5 days to discharge)** | | | | | | | | | | | | | | | | |
| Intense chills/shaking | X | X | X | X | X | X | X | X | X | X | X | X |  |  | X | X |
| Nausea/dry heaving | X | X | X | X | X | X | X | X | X |  | X | X |  | X |  | X |
| Fever/elevated temperature |  |  | X | X | X |  | X | X |  | X | X | X | X | X |  | X |
| Loss of appetite | X | X | X |  | X | X |  | X |  |  |  | X |  | X |  | X |
| Weakness/fatigue |  | X | X | X | X | X | X | X | X | X |  |  | X |  | X | X |
| Flank/stomach pain | X |  | X | X |  |  | X | X |  |  |  | X | X | X |  |  |
| Diarrhea |  | X |  | X | X |  | X | X | X |  |  | X |  |  | X |  |
| Shortness of breath/low oxygen levels |  |  |  | X | X |  |  | X |  | X | X | X |  |  | X |  |
| Tingling/burning during urination |  |  |  | X | X |  |  | X |  |  |  | X |  |  |  |  |
| Back pain |  |  |  |  |  |  |  |  |  |  | X | X |  |  |  |  |
| Low blood pressure symptoms |  | X |  |  | X |  |  |  |  |  |  |  |  |  |  |  |
| Headache |  |  | X |  |  |  |  | X | X |  |  |  |  |  |  |  |
| Lightheadedness |  | X |  |  |  |  |  | X |  |  |  |  |  |  |  | X |
| Inability to urinate/empty bladder |  |  |  |  |  |  | X | X |  | X |  |  | X |  |  | X |
| Constipation |  |  |  |  |  |  |  |  |  | X |  |  | X |  |  |  |
| Foul-smelling urine |  |  |  |  |  |  |  |  |  |  |  |  | X |  |  |  |
| Feeling of imbalance |  |  |  |  |  |  | X |  |  |  |  |  | X |  |  |  |
| Blood in urine |  |  |  |  |  |  |  | X |  |  |  |  | X | X |  | X |
| Frequent urination |  |  |  |  |  |  |  |  |  |  |  |  |  |  | X | X |
| **Ongoing symptoms *after* *hospital discharge* (discharge to interview [maximum 14 days])** | | | | | | | | | | | | | | | | |
| Weakness/fatigue | X | X |  | X | X | X | X | X | X |  |  | X |  |  |  |  |
| Diarrhea |  | X |  | X | X |  |  |  |  |  |  |  |  |  |  |  |
| Loss of appetite | X |  |  |  | X | X |  |  | X |  |  |  |  |  |  | X |
| Tingling/burning during urination |  |  |  | X |  |  |  |  |  |  |  | X |  |  |  |  |
| Stomach pain | X |  |  |  |  |  |  |  |  |  |  |  |  |  |  |  |
| Constipation |  |  |  |  |  |  |  |  |  |  |  |  | X |  |  |  |

Empty cell = not present

IED, invasive *Escherichia coli* disease

#### Table S3 IED impacts reported per study participant

| **IED case definition** | **Bacteremic** | | | | | | | | | | **Non-bacteremic** | | | | | |
| --- | --- | --- | --- | --- | --- | --- | --- | --- | --- | --- | --- | --- | --- | --- | --- | --- |
| **Participant number** | 1 | 4 | 5 | 6 | 7 | 8 | 11 | 12 | 13 | 14 | 2 | 3 | 9 | 10 | 15 | 16 |
| **Psychological impacts** | | | | | | | | | | | | | | | | |
| Confusion | X | X | X |  | X |  |  |  |  | X |  |  |  |  |  |  |
| Anxiety |  |  |  | X |  |  |  |  |  | X | X |  |  |  |  |  |
| Depression |  | X |  |  |  | X |  |  |  |  |  |  | X |  |  |  |
| Frustration |  |  |  |  |  | X |  | X |  |  |  |  |  |  |  |  |
| Disorientation | X |  | X |  | X |  |  |  |  |  |  |  |  |  |  |  |
| Fear | X | X |  | X | X | X |  |  |  | X | X |  |  |  |  |  |
| **Physical impacts** | | | | | | | | | | | | | | | | |
| Ability to engage in mobility | X | X | X | X | X |  | X | X | X |  | X | X |  |  | X |  |
| Impact on daily routines and activities | X |  | X | X | X | X | X | X | X | X | X | X |  | X | X |  |
| Not being able to sleep |  | X | X |  | X | X |  |  | X | X | X | X | X | X | X |  |
| Inability to communicate |  |  |  |  |  |  |  |  |  |  |  |  |  |  |  |  |
| Inability to feel balanced/imbalance | X |  | X |  |  |  |  |  |  |  |  | X |  |  | X |  |
| Learning to live with constant pain | X | X | X |  |  |  | X |  | X |  | X | X | X | X |  |  |
| Increased exhaustion |  | X | X | X | X | X |  |  |  |  | X | X |  | X | X | X |
| Not being able to concentrate |  |  | X |  |  |  |  |  |  |  |  |  |  |  |  | X |
| **Social impacts** | | | | | | | | | | | | | | | | |
| Engage in fewer activities | X | X | X | X | X | X | X |  | X |  | X | X | X | X | X | X |
| Temporarily rely on the support of caregiver/family member/friend |  | X | X |  |  |  |  |  |  |  |  | X |  |  |  |  |

Empty cell = not present

IED, invasive *Escherichia coli* disease
